# Difference of Liver and Kidney Metabolic Profiling in Chronic Atrophic Gastritis Rats between Acupuncture and Moxibustion Treatment

**DOI:** 10.1155/2018/6030929

**Published:** 2018-09-17

**Authors:** Qi-da He, Yuan-peng Huang, Li-bing Zhu, Jia-cheng Shen, Lin-yu Lian, Yuan Zhang, Long-bin Zhang, Lin-chao Qian, Xian-jun Meng, Mi Liu, Cai-chun Liu, Zong-bao Yang

**Affiliations:** ^1^Department of Traditional Chinese Medicine, Zhongshan Hospital and Shenzhen Research Institute, Xiamen University, Xiamen 361005, China; ^2^College of Acupuncture and Moxibustion, Fujian University of Traditional Chinese Medicine, Fuzhou 350122, China; ^3^College of Acupuncture and Moxibustion, Hunan University of Traditional Chinese Medicine, Changsha 410208, China

## Abstract

Acupuncture and moxibustion proved to be very effective in chronic atrophic gastritis (CAG). According to the Chinese traditional medicine theory, chronic diseases have an influence on the function of liver and kidney. However, there is little research to demonstrate this theory. This study is aimed at assessing the ^1^H NMR-based metabolic profiling in liver and kidney of CAG rats and comparing the difference between electroacupuncture and moxibustion treatment. Male SD rats were subjected to CAG modeling by intragastric administration of mixture of 2% sodium salicylate and 30% alcohol coupled with compulsive sporting and irregular fasting for 12 weeks and then treated by electroacupuncture or moxibustion at Liangmen (ST 21) and Zusanli (ST 36) acupoints for 2 weeks. A ^1^H NMR analysis of liver and kidney samples along with histopathological examination and molecular biological assay was employed to assess and compare the therapeutic effects of electroacupuncture and moxibustion. CAG brought characterization of metabolomic signatures in liver and kidney of rats. Both electroacupuncture and moxibustion treatment were found to normalize the CAG-induced changes by restoring energy metabolism, neurotransmitter metabolism, antioxidation metabolism, and other metabolism, while the moxibustion treatment reversed more metabolites related to energy metabolism in liver than electroacupuncture treatment. CAG did have influence on liver and kidney of rats. Both of these treatments had good effects on CAG by reversing the CAG-induced perturbation in liver and kidney. For regulating the energy metabolism in liver, the moxibustion played more important role than electroacupuncture treatment.

## 1. Introduction

Chronic atrophic gastritis (CAG) is a kind of gastrointestinal disorder and its clinical symptoms mainly include fullness, epigastric pain, anorexia, belching, and other nonspecific symptoms [1]. CAG has been shown to be a prime risk factor for the development of gastric cancer, which is the third leading cause of cancer-related death in the world [2, 3]. Although great progress has been made in management of CAG, most modern medicines, such as* Helicobacter pylori* (Hp) eradication, and synthetic medicine, remain unsatisfying because of side effects and high cost [4–6]. Therefore, an ideal strategy to relieve CAG is urgently needed.

Acupuncture and moxibustion, most popular complementary and alternative therapies, have been used for thousands of years in China [7]. Currently, they are increasingly used over time because of their lower costs and safety for clinical use [8]. Chinese traditional acupuncture stimulates acupuncture points by manual manipulations of needles, while electroacupuncture, as an improved version, delivers electrical pulses to needles and increases stimulation in order to improve the clinical effects [9]. Moxibustion stimulates certain acupoints with heat arising from burning moxa [10]. Recently, acupuncture and moxibustion have been shown to be effective in treating CAG [11, 12].

In addition, as the major components of the traditional Chinese medicine (TCM), acupuncture and moxibustion are used for treatment of different diseases based on TCM theory holding a holistic, dynamic, and natural view [13]. This specific feature is concordant with metabolomics, which simultaneously monitors and evaluates changes of metabolic profiles caused by changes in disease and other stimulations in a holistic context [14, 15]. Recently, ^1^H NMR-based based metabolomics has been extensively used in elucidation of the therapeutic mechanisms of CAG and its treatment because of nondestructive, minimal sample preparation and nonselective analysis [16–18]. In our previous study, we found that the electroacupuncture played an important role in CAG treatment mainly by regulating nervous system in stomach and brain, while moxibustion was effective in regulation of energy metabolism in serum by a ^1^H NMR-based metabolomics analysis of multiple biological samples (cerebral cortex, medulla, stomach, and serum) in CAG rats. According to the TCM theory, liver and the kidney are intimately related and chronic diseases have an influence on the function of liver and kidney [19].

In this study, the influence on CAG rats' liver and kidney and the difference between electroacupuncture and moxibustion treatment are investigated by ^1^H NMR-based metabolomics combined with pathological evaluation and molecular biological assay.

## 2. Materials and Methods

### 2.1. Materials

The following materials were used: moxa cones (height: 16 mm, diameter of inner hole: 2 mm, Han Medicine, Nanyang, China); 0.30 × 25 mm stainless steel acupuncture needle (Suzhou Acupuncture Goods Co., Ltd. Jiangsu, China); electroacupuncture apparatus (Model G6805-2; Qingdao Xinsheng Medical Instrument Factory, Shandong, China); Leica paraffin embedding station (EG 1160, Leica Biosystems Nussloch GmbH, Germany); Leica rotary microtome (RM 2135, Leica Biosystems Nussloch GmbH, Germany); TSP-d4 (Cambridge Isotope Laboratories, Inc., USA); K_2_HPO_4_, NaH2PO4·2H2O (Xilong Scientific Co., Ltd. Guangdong, China); D2O (633178, Sigma, USA); Anhydrous ethanol, 0.9% Sodium Chloride Injection, 4% Polyoxymethylene, 10% chloral hydrate (Changsha Guge Bio-Technology Co., Ltd., Changsha, China); NMR spectrometer (Bruker Biospin, Rheinstetten, Germany); NMR data preprocessing (MestReNova v9.0.1 software, Mestrelab Research, Santiago de Compostela, Spain); multivariate statistical analysis (SIMCA-P14.1, Umetrics, Sweden); ghrelin and substance P ELASA kit (Cusabio, Wuhan, Hubei, China).

### 2.2. Animals Handling

In this study, care and experimental procedures for all rats were approved by the Animal Care and Use Committee of Xiamen University (Permit Number: SCXK 2014-0001) and performed in accordance with National Institutes of Health ‘Guide for the Care and Use of Laboratory Animals'.

After a habituation for 7 days, all rats were randomly divided into six groups (n=6 each group): control group, chronic atrophic gastritis (CAG) group, electroacupuncture of the CAG rats on acupoints (EA group) as well as on nonacupoints (EN group), and moxibustion of the CAG rats on acupoints (MA group) and on nonacupoints (MN group). According to previous study [12], all rats except for controls were intragastrically administrated by mixture of 2% sodium salicylate and 30% alcohol and subjected to compulsive sporting as well as irregular fasting for 12 weeks.

### 2.3. Electroacupuncture and Moxibustion Treatment

According to the “Veterinary Acupuncture of China”, two acupuncture points including Zusanli (ST 36) and Liangmen (ST21) were selected (in Figure S1). Additionally, the nonacupoints were selected 5 mm away from each of two acupoints mentioned above and these nonacupoints do not lie on any other known acupoints and have nothing to do with the selected acupoints. After CAG modeling, the rats in EA group were subjected to electroacupuncture treatment on Zusanli (ST 36) and Liangmen (ST21) for 2 weeks (30-minute per day), while the rats in EN group were stimulated on the corresponding nonacupoints as mentioned above. Meanwhile, the rats in MA group and MN group were stimulated and subjected to moxibustion on the above-mentioned acupoints and corresponding nonacupoints (30 minutes per day) for two weeks, respectively.

According to previous study [12], an electroacupuncture apparatus coupled with stainless steel acupuncture needles and two-channel electrical stimulations at irregular waves (intermittent wave: 4 Hz; irregular wave: 50 Hz) with voltage (2~4 V) was used. During the moxibustion treatment, moxa cones were used (Figure S1).

### 2.4. Histopathology

The gastric mucosas from six groups' rats were collected and placed in phosphate-buffered 10% formalin. After dehydration, the biopsies were embedded in wax, sectioned at 5 *μ*m by Ultra-Thin Semiautomatic Microtome, and stained by hematoxylin and eosin. Finally, histopathological examination was conducted by light microscopy.

### 2.5. Enzyme Linked Immunosorbent Assay (ELISA) Assessment

All animals were anesthetized by inhaled isoflurane after electroacupuncture or moxibustion treatment for 14 days. The abdominal aorta blood of rats was collected. After standing at room temperature for 1 h, the serum samples were acquired by centrifuging (10000rmp, 10 min, 4°C). Each serum sample was divided into equal aliquots and stored at −80°C.

The ghrelin and substance P were determined by ELASA kit. The experience process was conducted according to the protocol provided by manufacturer.

### 2.6. Sample Collection and ^1^H NMR Experiments

All rats were sacrificed and anesthetized with isoflurane after the experimental procedure. The serum, liver, and kidney samples were collected and analyzed by NMR spectrometer as shown in the Supplementary Materials.

### 2.7. Data Preprocessing and Multivariate Statistical Analysis

Before being preprocessed via MestReNova v9.0.1 software, the ^1^H NMR spectral data was introduced into SIMCA-P14.1 for multivariate analysis as shown in the Supplementary Materials.

## 3. Results

### 3.1. Histological Morphology Examinations

**Figure 1:**
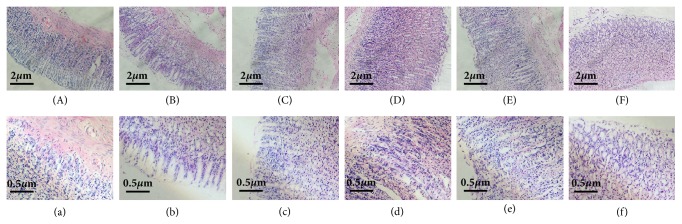
Histological examination of gastric mucosa from six groups. (A and a, the controls; B and b, the CAG group; C and c, EA group; D and d, EN group; E and e, MA group; F and f, MN group.) Scale bars represent 2 *μ*m for the top row and 0.5 *μ*m for the bottom row.

The histological morphology of gastric tissues of rats in the six groups was observed under microscope (in Figure 1). The intact histological structure of gastric tissue was shown and the sizes of epithelial cells and glands were well-arranged in control group. Besides, the cells were simple columnar and there were clear boundaries between epithelial and duct. On the contrary, for the CAG rats, thinning gastric mucosa layer and reduced gland cells were obviously seen, indicating that the CAG rats modeling was successful replication. Compared to the CAG group, the thickness of gastric mucosa and regular gland arrangement of rats in the EA group and MA group were improved in different degrees. This illustrates that both electroacupuncture and moxibustion on stomach meridian acupoints have significant curative effect on CAG rats. For rats in the EN group and MN group, the gastric epithelial cells were also improved but had a poorer effect than those in the EA group and MA group. In addition, the gastric mucosa thickness of rats in all group was investigated (Supplementary Table S1). And the results also showed that both electroacupuncture and moxibustion could improve the thickness of gastric mucosa in CAG rats effectively.

### 3.2. Examinations by Enzyme Linked Immunosorbent Assay (ELISA)

**Figure 2:**
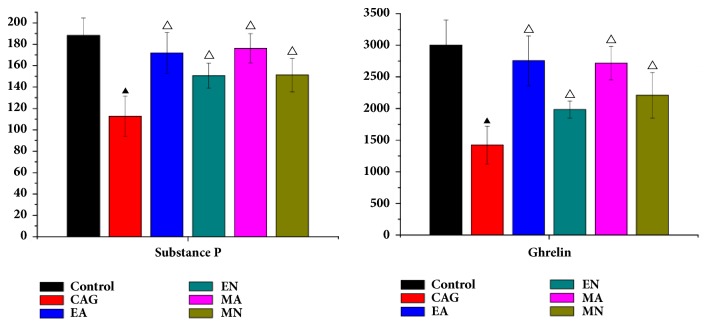
The expression of substance P and ghrelin in serum of rats in six groups. (▲ means a statistical significance p<0.05 when compared with the control group; △ means a statistical significance p<0.05 when compared with the CAG group.).

In this study, the levels of substance P (SP) and ghrelin in serum of all rats were detected by enzyme linked immunosorbent assay (ELISA) technology. Substance P and ghrelin in serum of CAG rats showed lower expression than that in control group. On the other hand, after electroacupuncture and moxibustion treatment, the levels of the two factors were increased significantly (in Figure 2).

### 3.3. ^1^H NMR Profiles of Liver and Kidney

**Figure 3:**
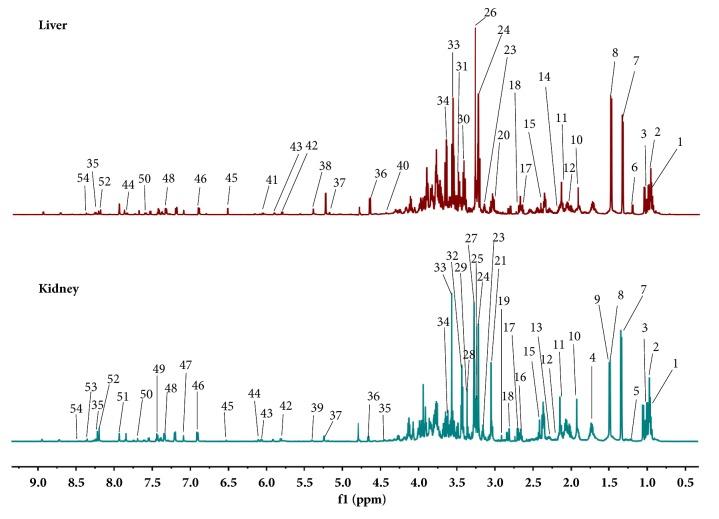
Typical 1H NMR spectra of extractive from liver and kidney (1, Isoleucine; 2, Leucine; 3, Valine; 4, Ornithine; 5, 3-Hydroxybutyrate; 6, Ethyl carbamate; 7, Lactate; 8, Alanine; 9, Lysine; 10, Acetate; 11, Glutamate; 12, Glutamine; 13, Methionine; 14, Glutathione; 15, Succinate; 16, Citrate; 17, Aspartate; 18, Dimethylamine; 19, Asparagine; 20, N-methylhydantoin; 21, Creatine; 22, Creatinine; 23, Ethanolamine; 24, Choline; 25, Phosphocholine; 26, Taurine; 27, Glycerophosphocholine; 28, Trimethylamine-N-oxide; 29, Taurine; 30, Betaine; 31, Inositol; 32, Scyllo-Inositol; 33, Glycine; 34, Glycerol; 35, Adenosine; 36, *β*-glucose; 37, *α*-glucose; 38, Glycogen; 39, Allantoin; 40, Uridine; 41, Inosine; 42, Uracil; 43, Uridine; 44, Cytidine; 45, Fumarate; 46, Tyrosine; 47, Histidine; 48, Tryptophan; 49, Phenylalanine; 50, Nicotinamide; 51, Xanthine; 52, Hypoxanthine; 53, Nicotinamide mononucleotide (NMN); 54, Formate; 55, Glucaric acid.).

The typical ^1^H NMR spectra of liver and kidney extracts are shown in Figure 3. To acquire a good metabolite identification, two-dimensional (2D) NMR spectra including ^1^H-^1^H correlation spectroscopy (COSY) and ^1^H-^13^C heteronuclear single quantum coherence (HSQC) for the samples were also conducted (Supplementary Figures S2-5). And the main metabolites from the spectra were identified according to our own developed NMR database and published references as well as Human Metabolome Database (HMDB: http://www.hmdb.ca/) (Supplementary Tables S2-3).

Visually, these ^1^H NMR spectra showed less obvious difference between groups because of the complexity of the spectra. To acquire any possible variables contributing to CAG molding as well as electroacupuncture or moxibustion treatment, all datasets were used to examine the clustering of each group by OPLS-DA. For liver and kidney, good separation between the control group and CAG group was clearly seen (Supplementary Figure S6), indicating an obvious change in metabolic profile of liver and kidney in CAG rats. To obtain metabolites related CAG modeling, the corresponding S-plot and t-test were also performed (Supplementary Figure S7).

**Figure 4:**
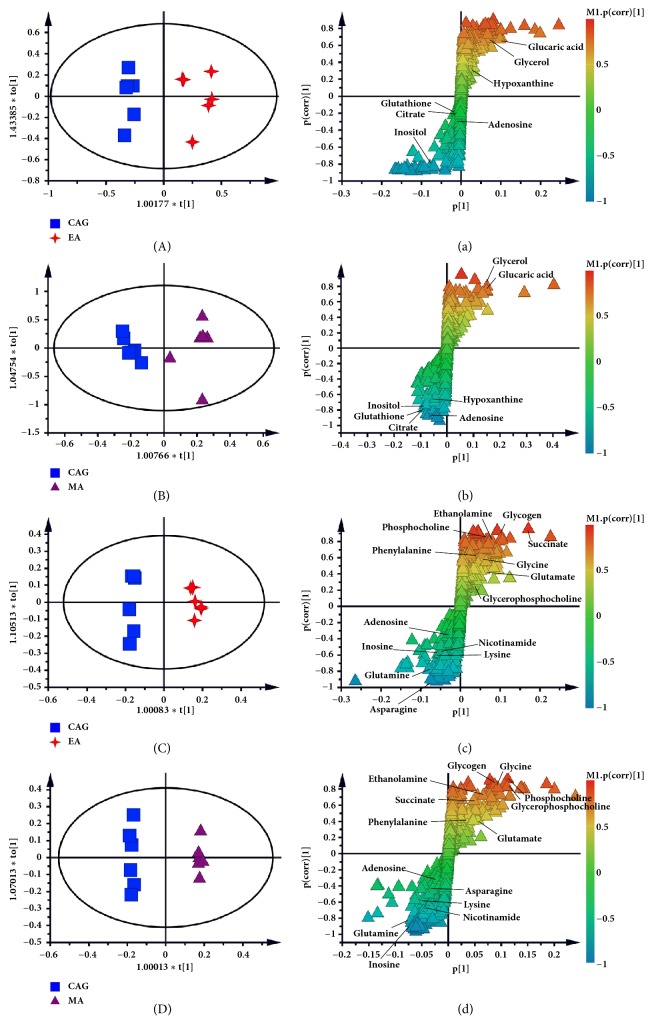
OPLS-DA scores plots and corresponding S-plots from the CAG and EA group in liver ((A) and (a), R2X (cum) =0.723, R2Y (cum) =0.933, Q2 (cum) =0.788) and in kidney ((C) and (c), R2X (cum) =0.395, R2Y (cum) =0.99, Q2 (cum) =0.908); OPLS-DA scores plots and corresponding S-plots from the CAG and MA group in liver ((B) and (b), R2X (cum) =0.874, R2Y (cum) =0.988, Q2 (cum) =0.858) and kidney ((D) and (d), R2X (cum) =0.48, R2Y (cum) =0.998, Q2 (cum) =0.934).

Using the strategy mentioned before, all of rats in EA or MA group presented clear separation from CAG rats (in Figure 4), indicating that both the electroacupuncture and moxibustion had a good effect on CAG rats. And the corresponding S-plots and the following t-test suggested the following: levels of Glycerol, Inositol, Glutathione, Glucaric acid, Succinate, Nicotinamide, Glutamate, Glycine, Glycogen, Phosphocholine, Glutamine, Inosine, Phenylalanine, and Ethanolamine were reversed by both electroacupuncture and moxibustion treatment. After the electroacupuncture treatment, the levels of Asparagine and Lysine were returned to normal, and the levels of Adenosine, Glycerophosphocholine, Citrate, Hypoxanthine, Adenosine, Glucaric acid, and Nicotinamide were regulated by only moxibustion treatment (Supplementary Figure S7).

## 4. Discussion

Brain-gut peptides (BGPs), a kind of neuropeptides and neuroendocrine, not only regulate the activities of central nervous system but also play an important role in the secretory and motor functions of the gastrointestinal tract [20, 21]. As the crucial member of BGPs, substance P and ghrelin are actively involved in most of the inflammatory pathways in gastrointestinal tracts and assist with the evaluation of the pathophysiology of gastrointestinal disease [22, 23]. In this study, these two factors were returned to normal levels by both electroacupuncture and moxibustion treatment, indicating that both of the treatments play an important role in CAG by regulating BGPs.

**Figure 5:**
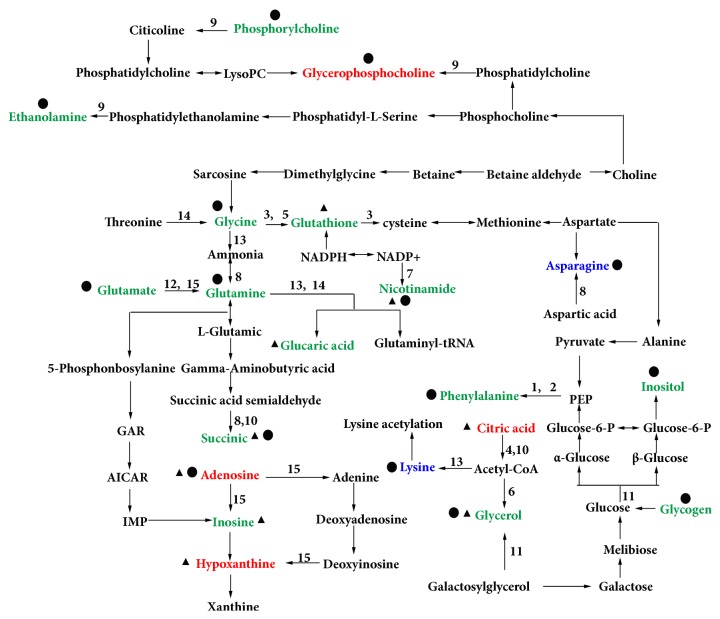
The metabolic pathways associated with electroacupuncture and moxibustion treatment on CAG rats. Metabolites in blue, red, and green represent metabolites regulated by electroacupuncture, moxibustion, and both of them, respectively. ▲ shows metabolites in liver; ● shows metabolites in kidney. (1, Phenylalanine, tyrosine, and tryptophan biosynthesis; 2, Phenylalanine metabolism; 3, Glutathione metabolism; 4, Glyoxylate and dicarboxylate metabolism; 5, Glycine, serine, and threonine metabolism; 6, Glycerolipid metabolism; 7, Nicotinate and Nicotinamide metabolism; 8, Alanine, aspartate, and Glutamate metabolism; 9, Glycerophospholipid metabolism; 10, Citrate cycle (TCA cycle); 11, Galactose metabolism; 12, D-Glutamine and D-Glutamate metabolism; 13, Lysine metabolism; 14, Aminoacyl-tRNA biosynthesis; 15, Purine metabolism.)

Additionally, the different levels of changes in metabolites in liver and kidney samples induced by CAG modeling were reversed by electroacupuncture or moxibustion treatment. These results may bring characteristic metabolomic profiles of CAG and the difference between moxibustion and electroacupuncture treatment, which will be discussed in further detail below (Figure 5 and Figure S8).

### 4.1. Energy Related Metabolism

Glucose is a primary source of energy for living organisms and is involved in a variety of energy metabolism pathways, such as gluconeogenesis and glycolysis [24]. Glycogen is the principal storage energy form of glucose in cell and Glucaric acid, a sugar acid derived from glucose [25, 26]. Inositol, synthesized from glucose, plays an essential role both physiologically and pathologically, while Glycerophosphocholine is an intracellular osmolyte and its content increasing in cells relies on the increased uptake of Inositol [27, 28]. Phosphocholine is the precursor metabolite in the glycerophospholipid metabolism pathway. Additionally, Citrate and Succinate play an important role in energy production as two of the dominant intermediates of tricarboxylic acid (TCA) cycle [29]. Interestingly, Lysine, through the transfer of an acetyl group from acetyl-CoA, can be converted to Lysine acetylation, which coordinately modulates glycolysis and the tricarboxylic acid (TCA) cycle [30]. Nicotinamide is a crucial component of the coenzyme NAD involved in glycolysis and the TCA cycle [31]. In addition, when the body used stored fat as a source of energy, Glycerol is released into the bloodstream and converted to glucose by the liver and provides energy for cellular metabolism [32]. On the other hand, Adenosine plays an important role in energy transfer as Adenosine triphosphate (ATP) as well as Adenosine diphosphate (ADP), and Hypoxanthine is a reaction intermediate in the metabolism of Adenosine [33]. Therefore, in this study, the levels of Glycogen, Glucaric acid, and Glycerol in liver and levels of Glycerophosphocholine, Glycogen, Phosphocholine, and Succinate in kidney of CAG rats are reversed to normal by both the electroacupuncture and moxibustion treatment, while the levels of Inositol, Citrate, Hypoxanthine, Adenosine, and Lysine in liver and Nicotinamide and Adenosine in kidney were regulated by only moxibustion treatment, which indicates that moxibustion may play a more important role in regulating energy metabolism than electroacupuncture treatment on CAG.

### 4.2. Neurotransmitter Related Metabolism

Asparagine is not an essential amino acid and is synthesized from oxaloacetate through a transaminase enzyme that transfers the amino group from Glutamate, which is the most abundant fast excitatory neurotransmitter in nervous system [34]. And Phenylalanine is a precursor of the neurotransmitters called catecholamines, which is another kind of neurotransmitter and acts as adrenalin-like substances [35]. For these two metabolites, Asparagine is regulated by only moxibustion treatment, while electroacupuncture treatment can reverse both of them to normal levels, suggesting that electroacupuncture treatment on CAG rats may have better effect in regulate neurotransmitter in nervous system than moxibustion treatment.

### 4.3. Antioxidant Related Metabolism

Glutathione is synthesized from cysteine, Glycine, and Glutamate and is also a powerful antioxidant because of its crucial thiol (-SH) group [36]. Additionally, it is reported that Glycine is an antioxidant and can mediate oxidative stress with activation of NMDA receptors in renal tissues of rats [37]. In current study, levels of these two metabolites in CAG rats were recovered to normal by both electroacupuncture and moxibustion treatment, indicating that both methods might treat CAG rats by regulating antioxidant related metabolism.

### 4.4. Other Metabolism

Ethanolamine is an important component of lecithin, and previous studies have reported that lecithin is helpful to improve the digestibility of fat [38]. Therefore, the lower levels of Ethanolamine in CAG rats' kidney returned to normal after electroacupuncture and moxibustion treatment in this present study, indicating that CAG could bring the fat indigestion and these two therapies are very effective approaches to treat CAG by improving fat indigestion. On the other hand, Inosine is transformed from Adenosine in the isolated basolateral membrane (BLM) of kidney proximal tubules by Adenosine deaminase and considered as one of the sensitive biomarkers to kidney injury [39]. In this study, the level of Inosine in CAG rats' kidney was lower than that of the controls and reversed to normal by electroacupuncture and moxibustion treatment. This result suggests that CAG does have an influence on kidney and both the two treatments on CAG play an important role in repairing the kidney.

Taken together, an obvious change in metabolic profile occurred in liver and kidney of CAG rats, involved in energy metabolism, neurotransmitter metabolism, antioxidation metabolism, and other metabolism. Both electroacupuncture and moxibustion intervention showed beneficial effects by restoring many CAG-induced metabolic changes. Notably, the moxibustion treatment plays more important role in regulating the energy metabolism in liver than electroacupuncture treatment. And electroacupuncture treatment on CAG rats has better effect in regulating neurotransmitter in nervous system than moxibustion treatment, while moxibustion plays a more important role in regulating energy metabolism. This finding will be beneficial for better understanding of difference in biological mechanism between electroacupuncture and moxibustion treatment on CAG.

## Data Availability

The original data (including MNOVA, Excel, and USP format) used to support the findings of this study were supplied by Zongbao Yang under license and so cannot be made freely available. Requests for access to these data should be made to ZongbaoYang, yangzb@xmu.edu.cn.

## Supplementary Materials

Supplementary Materials In order to provide complementary information, there are some materials shown in supplementary materials as follows. Figure S1. The location of acupoints and corresponding nonacupoints for Zusanli (ST 36, located 5 mm below the fibular head and lateral to the anterior tubercle of the tibia) on rats (a); the location of acupoints and corresponding nonacupoints for Liangmen (ST 21, located 5 mm horizontally to the spot above 2cm from navel) on rats (b); moxa cones (c); stainless steel acupuncture needles (d). Figure S2. Portion of the 1H−1H COSY 600 MHz spectrum of liver metabolites. Identified metabolites: 1, Isoleucine; 3, Valine; 5, 3-Hydroxybutyrate; 7, Lactate; 8, Alanine; 9, Lysine; 10, Acetate; 12, Glutamine; 23, Ethanolamine; 24, Choline; 30, Betaine; 31, Inositol; 35, Adenosine; 38, Glycogen; 40, Uridine; 41, Inosine; 43, Uridine; 44, Cytidine; 46, Tyrosine; 48, Tryptophan; 50, Nicotinamide. Figure S3. Portion of the 1H−13C HSQC 600 MHz spectrum of liver metabolites. Identified metabolites: 1, Isoleucine; 3, Valine; 5, 3-Hydroxybutyrate; 7, Lactate; 8, Alanine; 9, Lysine; 10, Acetate; 11, Glutamate; 12, Glutamine; 14, Glutathione; 15, Succinate; 18, Dimethylamine; 20, N-methylhydantoin; 21, Creatine; 23, Ethanolamine; 24, Choline; 25, Phosphocholine; 26, Taurine; 27, Glycerophosphocholine; 30, Betaine; 31, Inositol; 34, Glycerol; 35, Adenosine; 36, *β*-glucose; 37, *α*-glucose; 38, Glycogen; 41, Inosine; 43, Uridine. Figure S4. Portion of the 1H−1H COSY 600 MHz spectrum of kidney metabolites. Identified metabolites: 1, Isoleucine; 3, Valine; 4, Ornithine; 5, 3-Hydroxybutyrate; 7, Lactate; 8, Alanine; 9, Lysine; 17, Aspartate; 18, Dimethylamine; 23, Ethanolamine; 24, Choline; 30, Betaine; 46, Tyrosine; 48, Tryptophan; 50, Nicotinamide. Figure S5. Portion of the 1H−13C HSQC 600 MHz spectrum of kidney metabolites. Identified metabolites: 1, Isoleucine; 2, Leucine; 3, Valine; 4, Ornithine; 5, 3-Hydroxybutyrate; 7, Lactate; 8, Alanine; 9, Lysine; 10, Acetate; 12, Glutamine; 13, Methionine; 14, Glutathione; 16, Citrate (M); 17, Aspartate; 18, Dimethylamine; 21, Creatine; 24, Choline; 25, Phosphocholine; 30, Betaine; 31, Inositol; 33, Glycine; 34, Glycerol; 36, *β*-glucose; 37, *α*-glucose; 48, Tryptophan. Figure S6. OPLS-DA scores plots and corresponding S-plots from the CAG rats and the controls with electroacupuncture treatment on the stomach meridian acupoints in liver (A and a, R2X (cum) =0.848, R2Y (cum) =0.986, Q2 (cum) =0.925) and Kidney (B and b, R2X (cum) =0.573, R2Y (cum) =0.969, Q2 (cum) =0.61). Figure S7. Relative abundance (mean ± SD) of characteristic metabolites from serum and tissues in six groups (n=6). (Control, control rats; CAG, chronic atrophic gastritis rats; EA, CAG rats with electroacupuncture treatment on the stomach meridian acupoints; MA group, CAG rats with moxibustion treatment on the stomach meridian acupoints.) (▲ means a statistical significance p < 0.05 when compared with the control group; △ means a statistical significance p < 0.05 when compared with the CAG group.) Compared with the controls, some changes were shown in levels of metabolites in CAG rats as follows: a) for liver sample, levels of Citrate, Inositol, Glutathione, Hypoxanthine, and Adenosine were increased, whereas the levels of Glycerol and Glucaric acid were decreased; b) in kidney, the levels of Adenosine, Nicotinamide, Glutamine, Inosine, Asparagine, and Lysine were increased, while levels of Succinate, Glutamate, Glycine, Glycerophosphocholine, Glycogen, Phosphocholine, Phenylalanine, and Ethanolamine were decreased. Figure S8. The overview of metabolic pathways related to electroacupuncture and moxibustion treatment on CAG rats from KEGG. (1, Phenylalanine, tyrosine, and tryptophan biosynthesis; 2, Phenylalanine metabolism; 3, Glutathione metabolism; 4, Glyoxylate and dicarboxylate metabolism; 5, Glycine, serine and threonine metabolism; 6, Glycerolipid metabolism; 7, Nicotinate and Nicotinamide metabolism; 8, Alanine, aspartate, and Glutamate metabolism; 9, Glycerophospholipid metabolism; 10, Citrate cycle (TCA cycle); 11, Galactose metabolism; 12, Glutamine and Glutamate metabolism; 13, Lysine metabolism; 14, Aminoacyl-tRNA biosynthesis; 15, Purine metabolism.) Table S1. Gastric mucosa thickness of rats in the six groups (mean ± SD). Table S2. Peak attribution of the main marked metabolites in 1H-NMR spectra of liver sample. Table S3. 1H-NMR data and assignments of the metabolites in kidney aqueous extracts.
