# Supplementary material for: Difference of Liver and Kidney Metabolic Profiling in Chronic Atrophic Gastritis Rats between Acupuncture and Moxibustion Treatment
Source: Evid Based Complement Alternat Med. 2018 Sep 17;2018:6030929. doi: 10.1155/2018/6030929 (PMC6166372; doi:10.1155/2018/6030929)
Supplement: Supplementary Materials — In order to provide complementary information, there are some materials shown in supplementary materials as follows. Figure S1. The location of acupoints and corresponding nonacupoints for Zusanli (ST 36, located 5 mm below the fibular head and lateral to the anterior tubercle of the tibia) on rats (a); the location of acupoints and corresponding nonacupoints for Liangmen (ST 21, located 5 mm horizontally to the spot above 2cm from navel) on rats (b); moxa cones (c); stainless steel acupuncture needles (d). Figure S2. Portion of the 1H−1H COSY 600 MHz spectrum of liver metabolites. Identified metabolites: 1, Isoleucine; 3, Valine; 5, 3-Hydroxybutyrate; 7, Lactate; 8, Alanine; 9, Lysine; 10, Acetate; 12, Glutamine; 23, Ethanolamine; 24, Choline; 30, Betaine; 31, Inositol; 35, Adenosine; 38, Glycogen; 40, Uridine; 41, Inosine; 43, Uridine; 44, Cytidine; 46, Tyrosine; 48, Tryptophan; 50, Nicotinamide. Figure S3. Portion of the 1H−13C HSQC 600 MHz spectrum of liver metabolites. Identified metabolites: 1, Isoleucine; 3, Valine; 5, 3-Hydroxybutyrate; 7, Lactate; 8, Alanine; 9, Lysine; 10, Acetate; 11, Glutamate; 12, Glutamine; 14, Glutathione; 15, Succinate; 18, Dimethylamine; 20, N-methylhydantoin; 21, Creatine; 23, Ethanolamine; 24, Choline; 25, Phosphocholine; 26, Taurine; 27, Glycerophosphocholine; 30, Betaine; 31, Inositol; 34, Glycerol; 35, Adenosine; 36, β-glucose; 37, α-glucose; 38, Glycogen; 41, Inosine; 43, Uridine. Figure S4. Portion of the 1H−1H COSY 600 MHz spectrum of kidney metabolites. Identified metabolites: 1, Isoleucine; 3, Valine; 4, Ornithine; 5, 3-Hydroxybutyrate; 7, Lactate; 8, Alanine; 9, Lysine; 17, Aspartate; 18, Dimethylamine; 23, Ethanolamine; 24, Choline; 30, Betaine; 46, Tyrosine; 48, Tryptophan; 50, Nicotinamide. Figure S5. Portion of the 1H−13C HSQC 600 MHz spectrum of kidney metabolites. Identified metabolites: 1, Isoleucine; 2, Leucine; 3, Valine; 4, Ornithine; 5, 3-Hydroxybutyrate; 7, Lactate; 8, Alanine; 9, Lysine; 10, Acetate; 12, Glut [file 6030929.f1.zip › mat.6030929.v2.pdf]

Difference of liver and kidney metabolic profiling in chronic atrophic gastritis rats between acupuncture and moxibustion treatment

Qi-da He[1,2]; Yuan-peng Huang†[1]; Li-bing Zhu[1]; Jia-cheng Shen[1]; Lin-yu Lian[1,2]; Yuan Zhang[1,3]; Long-bin, Zhang[1]; Lin-chao Qian[1]; Xian-jun Meng[1]; Mi Liu[3]; Cai-chun Liu[1※]; Zong-bao Yang [1※]

[1]Department of Traditional Chinese Medicine, Zhongshan hospital and Shenzhen Research Institute, Xiamen University, Xiamen 361005, China

[2] College of Acupuncture and Moxibustion, Fujian university of Traditional Chinese Medicine, Fuzhou 350122, China

[3] College of Acupuncture and Moxibustion, Hunan university of Traditional Chinese Medicine, Changsha 410208, China

[*]Correspondence and requests for materials should be addressed to Zong-bao Yang （ yzbldq@163.com.cn）and Cai-chun Liu (caichunliu714@g mail.com)

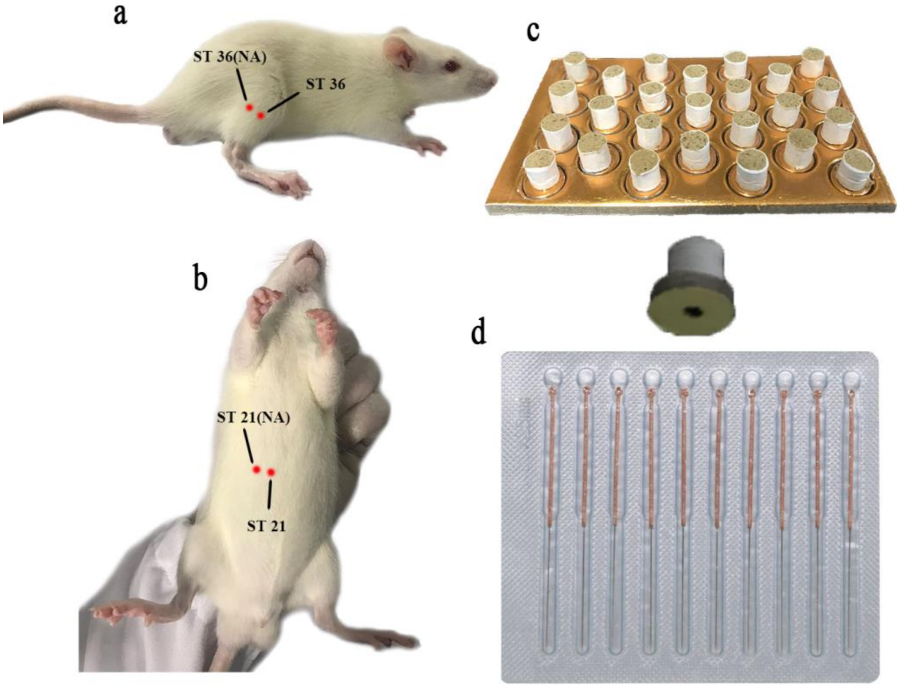

Figure.S1 The location of acupoints and corresponding non-acupoints for Zusanli (ST 36, located 5 mm below the fibular head and lateral to the anterior tubercle of the tibia) on rats (a); The

location of acupoints and corresponding non-acupoints for Liangmen (ST 21, located 5 mm horizontally to the spot above 2cm from navel) on rats(b); moxa cones(c); tainless-steel acupuncture needles(d).

Table.S1 Gastric mucosa thickness of rats in the six groups (mean ±S.D).

Group	Control	CAG	EA	EN	MA	MN
mean ±S.D	587.59±19.59	307.00±11.82▲	497.09±14.08△	423.07±75.35	524.73±47.79△	449.06±39.62△

[a] compare to the controls, the thickness of the gastric mucosa of CAG rats was thinner significantly (p<0.05), indicating the CAG modeling was successfully replicated. In addition, for rats in EA group, MA group and MN group, the thickness of gastric mucosa was thicker than that of CAG rats (p<0.05), while the thickness of gastric mucosa of rats in EN group has no significant difference, illustrating that both electro-acupuncture and moxibustion could improve the thickness of gastric mucosa in CAG rats effectively. (▲ indicates a statistical significance p<0.05 when compared with the control group. △ indicates a statistical significance p<0.05 when compared with the CAG group)

Each of liver and kidney tissue sample (weighed 200 mg) were homogenated in mixture of 300 mL H_2O and 600 mL CH_3OH and then vortexed for 1min. After partitioning on ice for 10 min, the samples were centrifuged for 10 min (10000 rpm, 4℃). The upper supernatant from each sample was lyophilized and mixed with 600 μL D_2O containing sodium 3-trimethylsilyl-(2, 2, 3, 3-d4)-1-propionate (TSP, 1 mM). Finally, a 500 μL of mixture was collected into 5 mm NMR tube for NMR analysis. For better metabolite identification, the two-dimensional (2D) NMR spectra including including ^{1}H-^{1}H correlation spectroscopy (COSY) and ^{1}H-^{13}C heteronuclear single quantum coherence (HSQC) for the liver and kidney sample was also captured. ^{1}H NMR spectra of all samples were acquired by a Bruker 600 MHz spectrometer at 298 K. 1D ^{1}H spectra were conducted with a Nuclear Overhauser Effect Spectroscopy (NOESY, RD-901-t1-90º-tm-90º-acquire) pulse sequence. For all samples, 64 FIDs were collected into 64K data points over a spectral width of 12 000 Hz with a relaxation delay of 6.5 μs. The COSY spectra was obtained with a relaxation delay of 1.5s, and 48 transients were collected into 1024 data points with a

spectral width of 12 ppm for both dimensions. In 2D ^{1}H-^{13}C HSQC spectra, a 1.5 s relaxation delay was used for water suppression by presaturation. Additionally, for every 2D spectrum, 2048×128 data points were collected using 8 scans per increment. The spectral widths were set to 12 and 240 ppm in the proton and carbon dimensions, respectively.

The metabolites in NMR spectra from all samples were assigned according to published literatures, [12,18] our own developed NMR database and HMDB database (http://www.hm db.ca/).

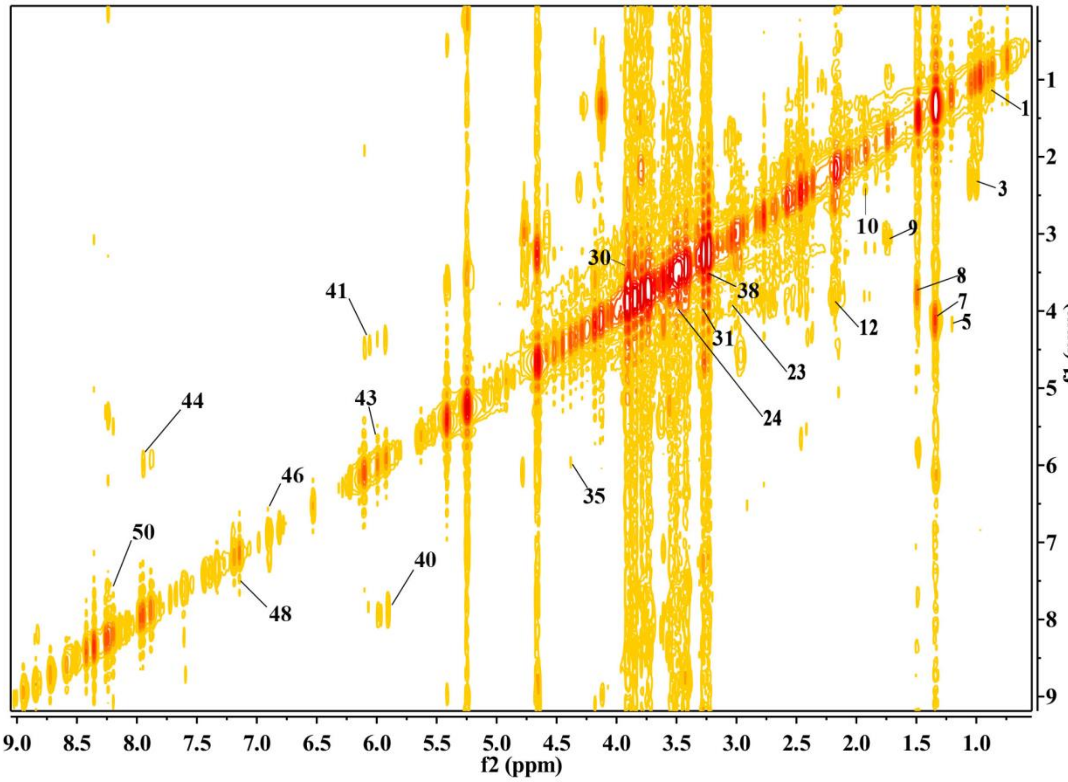

Figure.S2. Portion of the ^{1}H–^{1}H COSY 600 MHz spectrum of liver metabolites. Identified metabolites: 1, Isoleucine; 3,Valine; 5,3-Hydroxybutyrate; 7,Lactate; 8,Alanine;9, Lysine; 10,Acetate; 12,Glutamine; 23,Ethanolamine; 24,Choline; 30,Betaine; 31, Inositol; 35,Adenosine; 38,Glycogen; 40,Uridine; 41,Inosine; 43,Uridine; 44,Cytidine; 46,Tyrosine; 48,Tryptophan; 50, Nicotinamide.

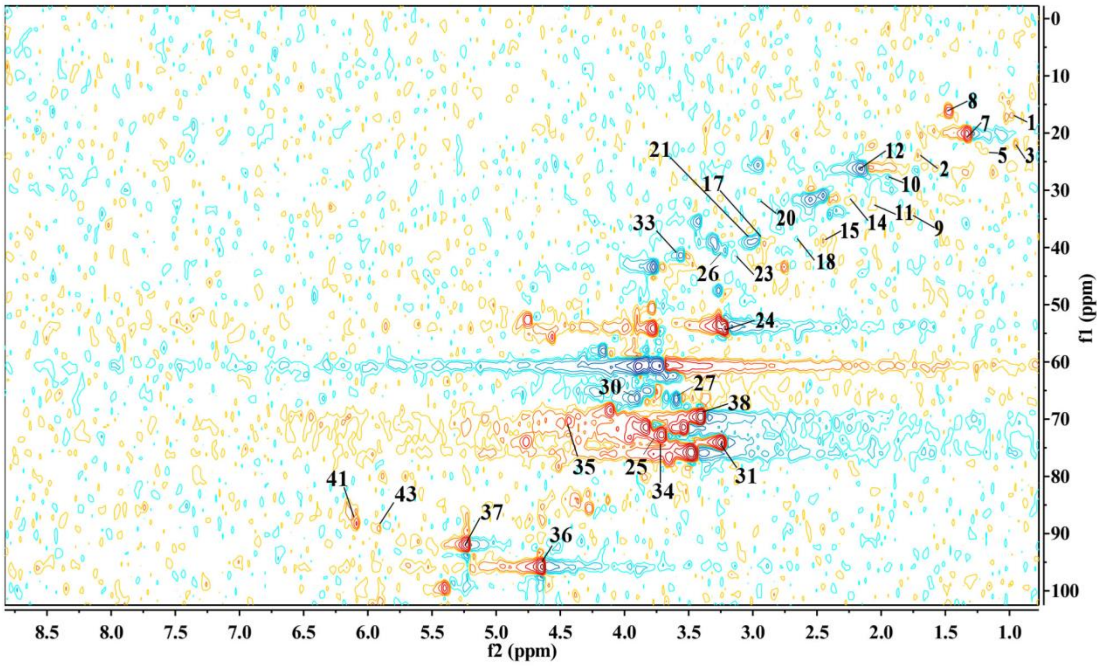

Figure.S3. Portion of the $^{1}H-^{13}C$ HSQC 600 MHz spectrum of liver metabolites. Identified metabolites: 1, Isoleucine; 3,Valine; 5,3-Hydroxybutyrate; 7, Lactate; 8, Alanine; 9, Lysine; 10, Acetate; 11, Glutamate; 12, Glutamine; 14, Glutathione; 15, Succinate; 18, Dimethylamine; 20, N-methylhydantoin; 21, Creatine; 23,Ethanolamine; 24,Choline; 25, Phosphocholine; 26,Taurine; 27,Glycerophosphocholine; 30, Betaine; 31, Inositol; 34,Glycerol; 35,Adenosine; 36,β-glucose; 37,α-glucose; 38,Glycogen; 41,Inosine; 43,Uridine.

Table S2 Peak attribution of the main marked metabolites in ^{1}H-NMR spectra of liver sample

NO.	Metabolites	δ1H/ppm	Moieties	δ^{13}C/pm	Assigned with
1	Isoleucine	0.94(t); 1.01(d)	δ-CH_3; β-CH3	17.13	COSY,HSQC
2	Leucine	0.96(t); 1.70(m)	CH_3; CH_2&γ-CH	23.57	HSQC
3	Valine	0.99(d); 1.04(d)	γ-CH_3; γ-CH'_3	21.77	COSY,HSQC
5	3-Hydroxybutyrate	1.21(d)	γCH_3	23.09	COSY,HSQC
7	Lactate	1.33(d); 4.11(q)	CH_3; CH	20.41	COSY,HSQC
8	Alanine	1.48(d); 3.78(q)	CH_3; CH	18.03	COSY,HSQC
9	Lysine	1.73(m), 3.02(t)	βCH_2,δCH_2	32.37	COSY,HSQC
10	Acetate	1.92(s)	CH_3	26.41	HSQC
11	Glutamate	2.05(m)	β-CH	32.87	HSQC
12	Glutamine	2.13(m),3.77(t)	βCH_2,γCH_2	26.75	COSY,HSQC

**14**	Glutathione	2.16 (m),2.55 (m)	β-CH_2; γ-CH_2	31.32	HSQC
**15**	Succinate	2.41(s)	CH	38.02	HSQC
**18**	Dimethylamine	2.72 (s)	CH_3	37.92	HSQC
**20**	N-methylhydantoin	2.92 (s),4.08 (s)	CH_3,CH_2	31.02	HSQC
**23**	Ethanolamine	3.13 (d)	CH_2	42.09	HSQC
**24**	Choline	3.20(s);3.52(m);4.07(m)	CH_3;N-CH_2;O-CH_2	54.92	HSQC
**25**	Phosphocholine	3.22(s);3.59(m);	CH3; N-CH_2;	72.25	HSQC
**26**	Phosphoethanolamine	3.23(t);	NCH_2;	40.26	HSQC
**27**	Glycerophosphocholine	3.68(m);	N-CH_2&HO-CH_2;	65.69	HSQC
**30**	Betaine	3.27(s); 3.89(s)	CH_3; CH_2	65.56	COSY,HSQC
**31**	Inositol	3.28(t);3.54(dd);	CH(2); CH(4, 6);	74.45	COSY, HSQC
**33**	Glycine	3.56 (s)	CH2	42.20	HSQC
**34**	Glycerol	3.64 (m)；3.77 (m)	CH2；CH	73.36	HSQC
**35**	Adenosine	4.45(t),6.10(d),8.25(s),8.35(s)	3-C’H,1-C’H,8-CH, 2-CH	70.79	COSY, HSQC
**36**	β-glucose	4.63 (d)	1-CH	95.28	HSQC
**37**	α-glucose	5.23 (d)	1-CH	92.31	HSQC
**38**	Glycogen	3.40(m)	1-CH	69.58	COSY, HSQC
**39**	Allantoin	5.39(s)	CH		
**40**	Uridine	5.91(d);7.87(d)	CH(2);CH(11)		COSY
**41**	Inosine	6.09 (d)	1-CH	90.18	COSY, HSQC
**42**	Uracil	5.80(d); 7.53(d)	CH(5); CH(6)		
**43**	Guanosine	5.90(s);	CH(2);	89.16	COSY, HSQC
**44**	Cytidine	6.06(d); 7.84(d)	CH(2); CH(11)		COSY
**45**	Fumarate	6.52s)	CH		
**46**	Tyrosine	6.89(d); 7.19(d)	m-CH; o-CH		COSY
**48**	Tryptophan	7.19(m);7.31(s);7.60(m);	CH(8);CH(6);CH(7)		COSY
**50**	Nicotinamide	7.59(dd);8.24(dd);8.72(dd); 8.94(s)	CH(5);CH(4);CH(6); CH(2)		COSY

**52**	Hypoxanthine	8.19(s); 8.21(s)	CH(2); CH(7)
**54**	Formate	8.46(s)	CH
**55**	Glucaric acid	3.95(t)	CH(8)

[a] Multiplicity for ^{1}H resonances: s: single; d: doublet; t: triplet; q: quartet; m: multiplet.

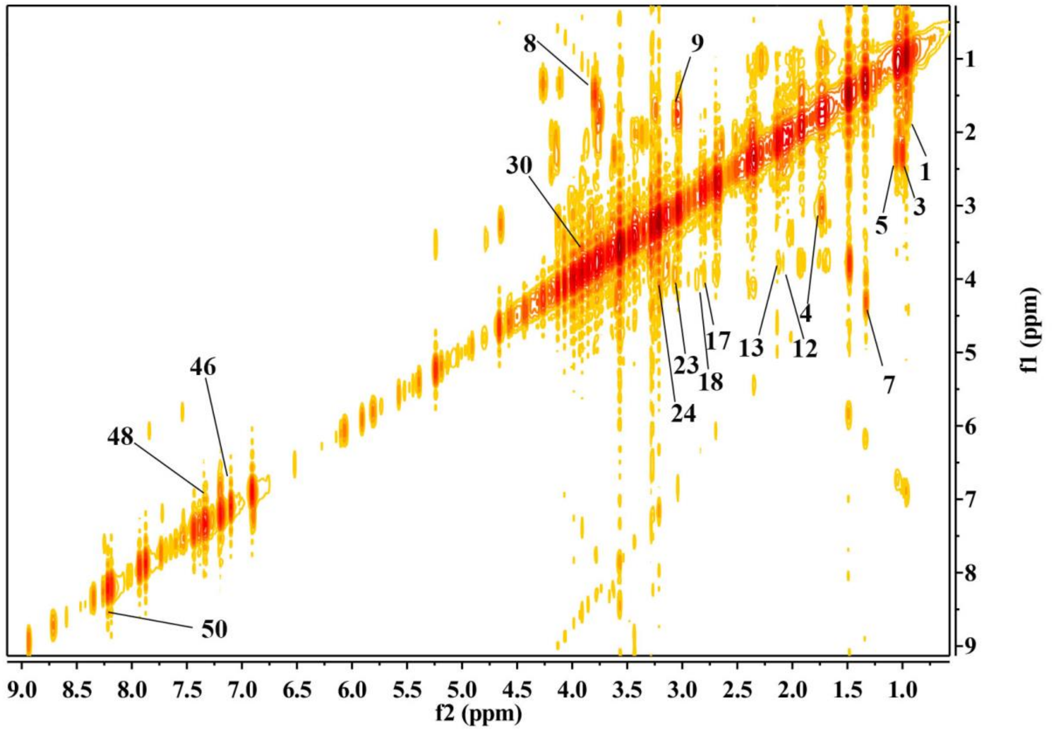

Figure.S4. Portion of the ^{1}H–^{1}H COSY 600 MHz spectrum of kidney metabolites. Identified metabolites:1, Isoleucine; 3,Valine; 4,Ornithine; 5,3-Hydroxybutyrate; 7,Lactate; 8,Alanine;9, Lysine; 17,Aspartate; 18,Dimethylamine; 23,Ethanolamine; 24,Choline; 30,Betaine; 46,Tyrosine; 48,Tryptophan; 50, Nicotinamide.

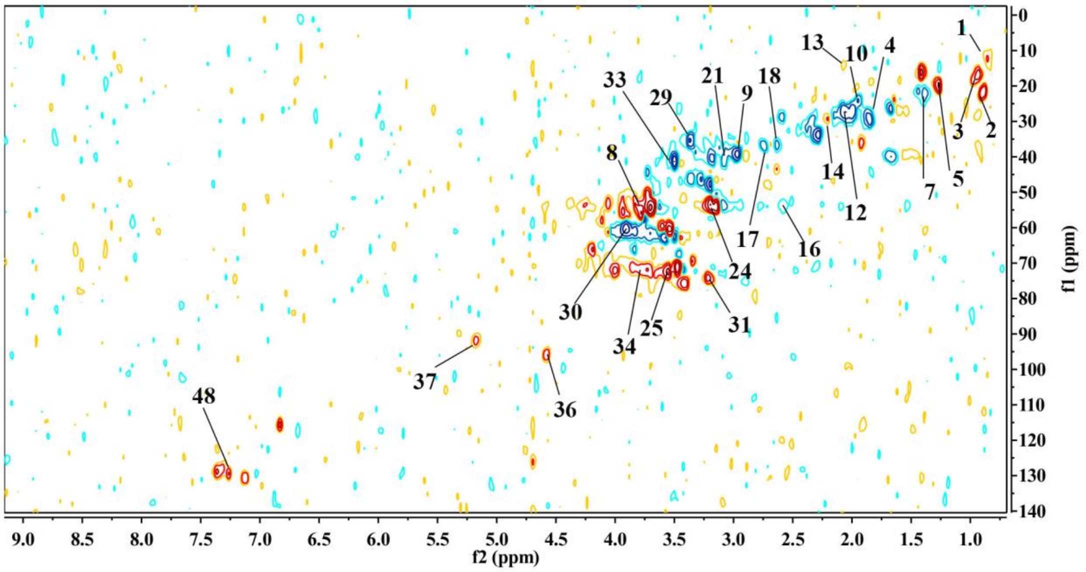

Figure.S5. Portion of the ^{1}H–^{13}C HSQC 600 MHz spectrum of kidney metabolites. Identified metabolites: 1, Isoleucine; 2,Leucine; 3,Valine; 4,Ornithine; 5,3-Hydroxybutyrate; 7,Lactate; 8,Alanine;9, Lysine; 10,Acetate; 12,Glutamine; 13,Methionine; 14,Glutathione; 16,Citrate (M); 17,Aspartate; 18,Dimethylamine; 21,Creatine; 24,Choline; 25,Phosphocholine; 30,Betaine; 31, Inositol; 33,Glycine; 34,Glycerol; 36,β-glucose; 37,α-glucose; 48,Tryptophan.

Table.S3 ^{1}H-NMR data and assignments of the metabolites in kidney aqueous extracts

NO.	Metabolites	δ1H/ppm	Moieties	$\delta^{13}C$/ppm	Assigned with
**1**	Isoleucine	0.94(t)	δ-CH_3	13.81	COSY,HSQC
**2**	Leucine	0.96(t);1.70(m)	CH_3;CH2&γ-CH	25.51	HSQC
**3**	Valine	0.99(d);1.04(d)	γ-CH3;γ-CH 3	22.90	COSY,HSQC
**4**	Ornithine	1.73(m)	δCH2	25.81	COSY,HSQC
**5**	3-Hydroxybutyrate	1.21(d)	γCH_3	21.40	COSY,HSQC
**7**	Lactate	1.33(d); 4.11(q)	CH_3; CH	20.27	COSY,HSQC
**8**	Alanine	1.48(d); 3.78(q)	CH_3;CH	54.58	COSY,HSQC
**9**	Lysine	1.73(m); 3.02(t)	βCH_2;δCH_2	39.93	COSY,HSQC
**10**	Acetate	1.92(s)	CH_3	25.83	HSQC
**11**	Glutamate	2.05(m)	β-CH		
**12**	Glutamine	2.13(m);3.77(t)	βCH_2;γCH_2	28.28	COSY,HSQC
**13**	Methionine	2.14(s) ;2.65(t)	γCH_2;S-CH_3	14.58	COSY,HSQC

**14**	Glutathione	2.16 (m);2.55 (m)	β-CH_2; γ-CH_2	29.58	HSQC
**15**	Succinate	2.41(s)	CH		
**16**	Citrate (M)	2.54(d);2.67(d)	CH_2;CH_2	53.55	HSQC
**17**	Aspartate	2.69(dd);2.82(dd)	βCH_2;βCH_2	35.95	COSY,HSQC
**18**	Dimethylamine	2.72 (s)	CH_3	36.69	HSQC
**19**	Asparagine	2.88(dd);2.96(dd)	βCH_2;βCH_2		COSY
**21**	Creatine	3.04(s);3.94(s)	CH_3,CH_2	38.55	HSQC
**22**	Creatinine	3.05(s);4.06(s)	CH_3;CH_2		
**23**	Ethanolamine	3.13 (d)	CH_2		COSY
**24**	Choline	3.20(s);3.52(m);4.07(m)	CH_3;N-CH_2;O-CH_2	53.19	COSY ,HSQC
**25**	Phosphocholine	3.22(s);3.59(m)	CH3; N-CH_2;	71.97	HSQC
**27**	Glycerophosphocholine	3.68(m)	N-CH_2&HO-CH_2		
**28**	Trimethylamine-N-oxide	3.27(s)	CH_3		
**29**	Taurine	3.27(t);3.42(t)	S-CH_2;N-CH_2	35.95	HSQC
**30**	Betaine	3.27(s); 3.89(s)	CH_3; CH_2	61.34	COSY,HSQC
**31**	Inositol	3.28(t);3.54(dd)	CH(2); CH(4, 6);	75.04	HSQC
**32**	Scyllo-Inositol	3.37(s)	CH		
**33**	Glycine	3.56 (s)	CH_2	43.27	HSQC
**34**	Glycerol	3.64 (m)；3.77 (m)	CH_2;CH	72.44	HSQC
**35**	Adenosine	6.10(d);8.25(s);8.35(s)	1-C'H;8-CH;2-CH		
**36**	β-glucose	4.63 (d)	1-CH	96.41	HSQC
**37**	α-glucose	5.23 (d)	1-CH	92.16	HSQC
**39**	Allantoin	5.39(s)	CH		
**40**	Uridine	5.91(d);7.87(d)	CH(2);CH(11)		
**42**	Uracil	5.80(d); 7.53(d)	CH(5); CH(6)		
**43**	Guanosine	5.90(s);	CH(2);		
**44**	Cytidine	6.06(d); 7.84(d)	CH(2); CH(11)		
**45**	Fumarate	6.52s)	CH		
**46**	Tyrosine	6.89(d); 7.19(d)	m-CH; o-CH		COSY

**47**	Histidine	7.11(s), 7.92(s)	2-CH, 4-CH		
**48**	Tryptophan	7.19(m);7.31(s);	CH(8); CH(6);	129.55	HSQC
		7.60(m);	CH(7);		
**49**	Phenylalanine	7.38(m),7.43(m)	,3,5-CH,4-CH		
**50**	Nicotinamide	7.59(dd);8.24(dd);	CH(5);CH(4);CH(6)		COSY
		8.72(dd); 8.94(s)	; CH(2)		
**51**	Xanthine	7.93(s)	CH		
**52**	Hypoxanthine	8.19(s); 8.21(s)	CH(2); CH(7)		
**53**	NMN	8.31(t); 9.00(d)	6-CH;2-CH		
**54**	Formate	8.46(s)	CH		

[a] Multiplicity for ^{1}H resonances: s: single; d: doublet; t: triplet; q: quartet; m: multiplet.

[b] NMN: nicotinamide mononucleotide

All of ^{1}H NMR spectra of tissue samples were phased and baseline corrected by MestReNova v9.0.1 software (Mestrelab Research, Santiago de Compostella, Spain). In ^{1}H NMR spectra, a single peak from TSP at 0.0 ppm was referenced. In order to overcome peak-shift problem, all spectra were also peak-aligned. After removing the chemical shift range of δ 4.70-5.2 ppm to exclude the influence of water, the spectra were segmented across the region of 0.5-9.0 ppm at δ 0.01 intervals. To compensate for significant concentration differences between samples, the integral values from every spectrum were normalized to a sum of all of integrals in a spectrum, and then the date matrices were formed for further multivariate analysis.

The ^{1}H NMR spectral data was introduced into SIMCA-P14.1 (Umetrics, Sweden) for multivariate analysis. Prior to multivariate analysis, the Pareto-scaling was used to reduce the influence of artefacts and noise in the models. In this study, a principal component analysis (PCA) was firstly conducted for a natural separation among all groups by visual inspection of the score plots. And then supervised orthogonal projection to latent structures discriminant analysis (OPLS-DA) was used to maximize difference between groups. Meanwhile, the quality of OPLS-DA model was assessed by parameters for model fitness (R2) and predictive ability (Q2). To acquire the potential variables for differentiation, the corresponding S-plot of OPLS-DA model was conducted. Finally, according to the variable importance in the project (VIP≥1.00) of the

established OPLS-DA model and an independent-sample t-test ($p<0.05$) using (OriginProver.8.1), the potential biomarkers were extracted.

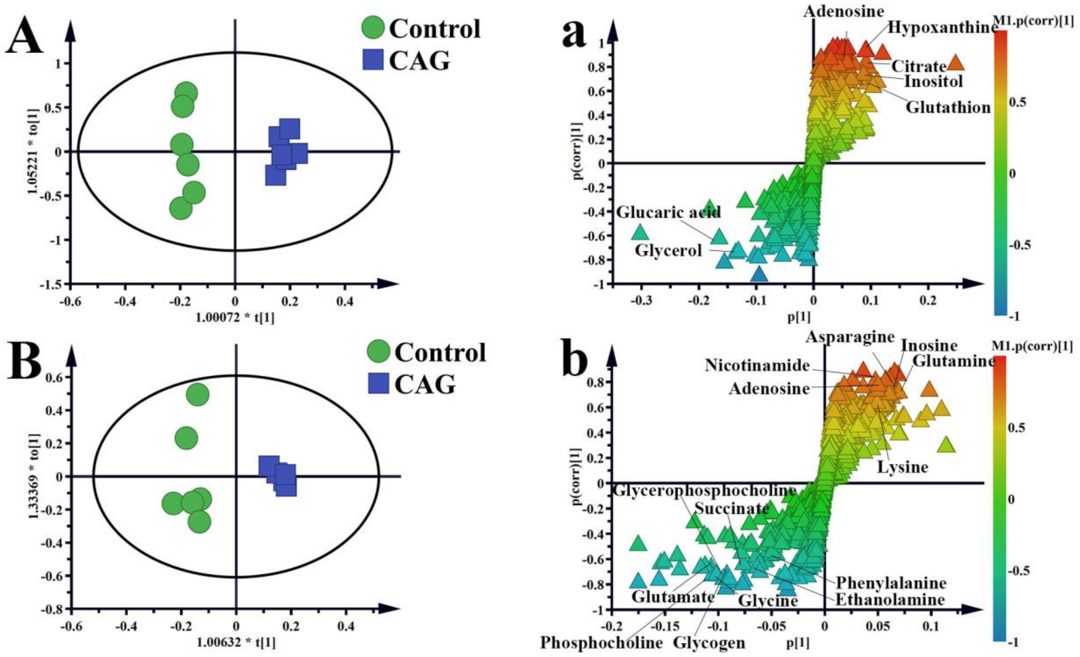

Figure.S6 OPLS-DA scores plots and corresponding S-plots from the CAG rats and the controls with electro-acupuncture treatment on the stomach meridian acupoints in Liver (A and a, R2X (cum) =0.848, R2Y(cum) =0.986, Q2(cum) =0.925)and Kidney (B and b, R2X (cum) =0.573, R2Y(cum) =0.969, Q2(cum) =0.61).

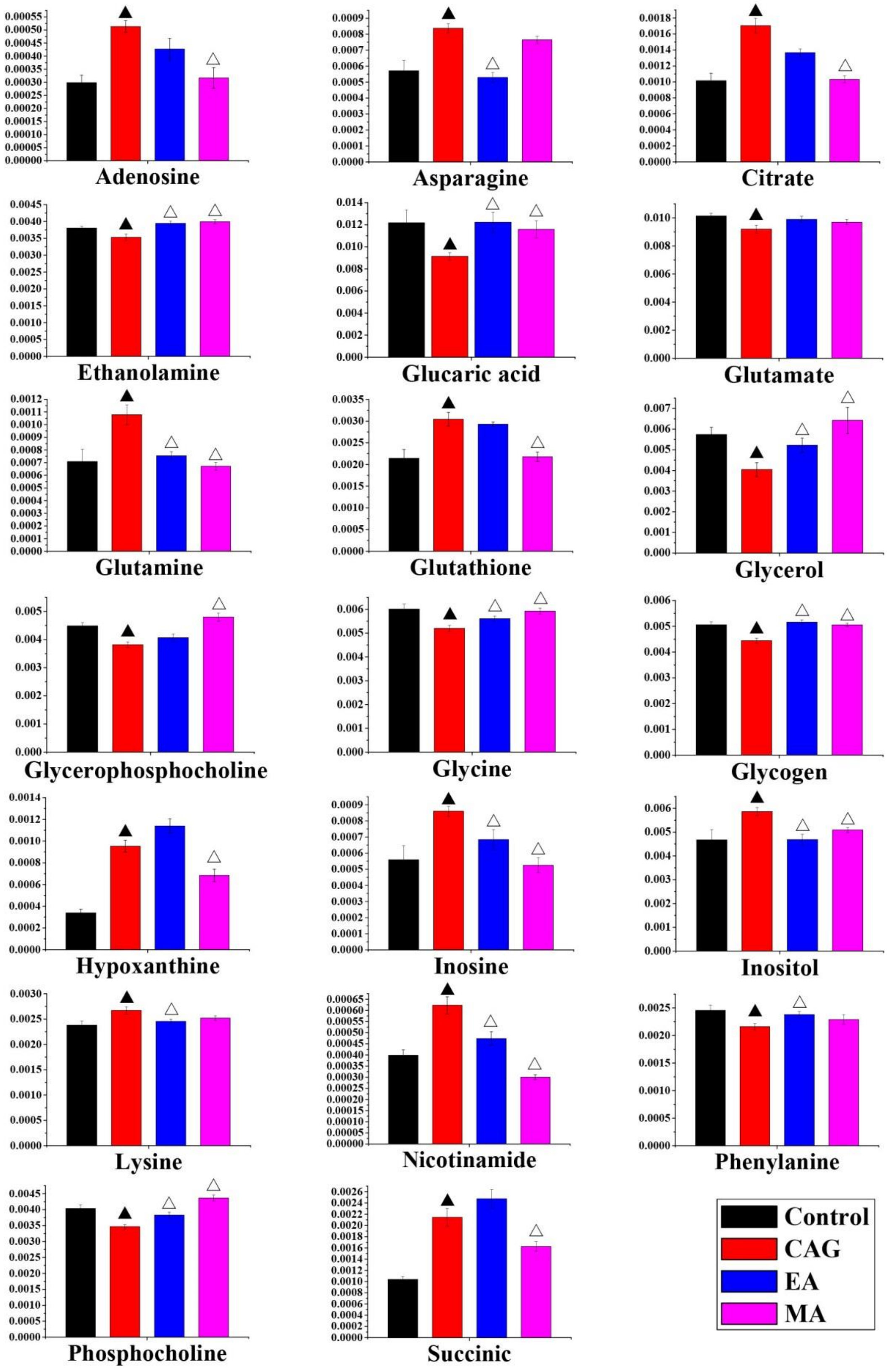

Figure.S7 Relative abundance (mean ± S.D.) of characteristic metabolites from serum and tissues in six groups (n=6). (Control, control rats; CAG, chronic atrophic gastritis rats; EA, CAG rats with electro-acupuncture treatment on the stomach meridian acupoints; MA group, CAG rats with

moxibustion treatment on the stomach meridian acupoints.) (▲ means a statistical significance $p < 0.05$ when compared with the control group; △ means a statistical significance $p < 0.05$ when compared with the CAG group.) Compared with the controls, It was showed some changes in levels of metabolites in CAG rats as follows: a) for liver sample, levels of citrate, inositol, glutathione, hypoxanthine and adenosine were increased, whereas the levels of glycerol and glucaric acid were decreased. b) in kidney, the levels of adenosine, nicotinamide, glutamine, inosine, asparagine and lysine were increased, while levels of succinate, glutamate, glycine, glycerophosphocholine, glycogen, phosphocholine, phenylalanine and ethanolamine were decreased.

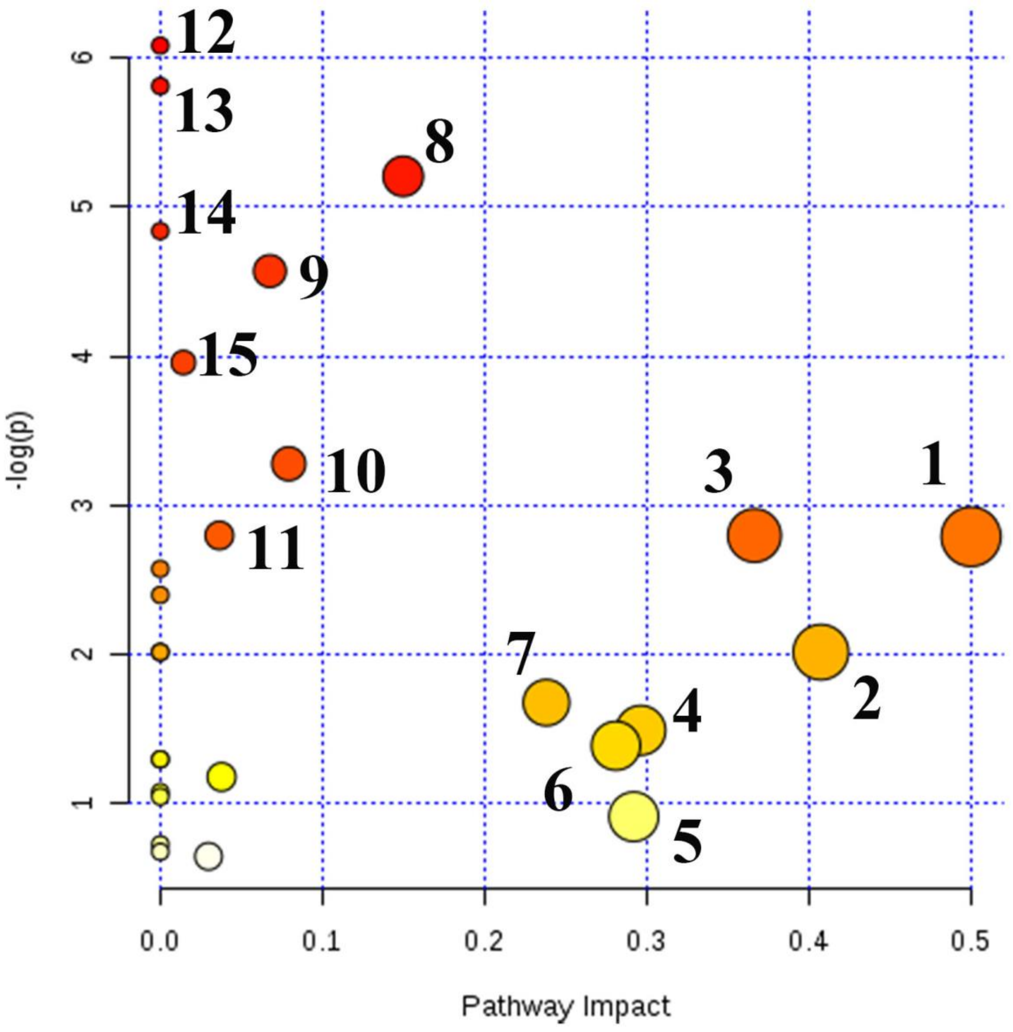

Figure.S8 The overview of metabolic pathways related to electro-acupuncture and moxibustion treatment on CAG rats from KEGG. (1,Phenylalanine, tyrosine and tryptophan biosynthesis; 2,

Phenylalanine metabolism; 3, Glutathione metabolism; 4,Glyoxylate and dicarboxylate metabolism; 5,Glycine, serine and threonine metabolism; 6, Glycerolipid metabolism; 7, Nicotinate and nicotinamide metabolism; 8, Alanine, aspartate and glutamate metabolism; 9, Glycerophospholipid metabolism; 10,Citrate cycle (TCA cycle);11,Galactose metabolism; 12, Glutamine and glutamate metabolism; 13, lysine metabolism; 14, Aminoacyl-tRNA biosynthesis; 15, Purine metabolism.)
